# Omic technology to monitoring resilience and adaptation to exercise and heat stress in endurance horses

**DOI:** 10.3389/fvets.2025.1734969

**Published:** 2026-01-09

**Authors:** Samanta Mecocci, Elisabetta Porzio, Elisabetta Chiaradia, Marco Pepe, Angelo Paris, Stefania Bergagna, Daniele Pietrucci, Giovanni Chillemi, Francesca Beccati, Katia Cappelli

**Affiliations:** 1 Department of Veterinary Medicine, University of Perugia, Perugia, Italy; 2 Department for Innovation in Biological, Agro-Food and Forest Systems (DIBAF), University of Tuscia, Viterbo, Italy; 3 Sports Horse Research Center, Department of Veterinary Medicine, University of Perugia, Perugia, Italy; 4 Private Practitioner, Sigillo, Italy; 5 Istituto Zooprofilattico Sperimentale del Piemonte Liguria e Valle d'Aosta (IZSPLV), Torino, Italy; 6 Department of Experimental Medicine, University of Rome “Tor Vergata,” Rome, Italy; 7 Bioinformatics Research Unit in Infectious Diseases, National Institute for Infectious Diseases Lazzaro Spallanzani - IRCCS, Rome, Italy

**Keywords:** field standardized exercise test, circulating miRNAs, liquid biopsy, personalized training, climate change, enviromental stress, welfare

## Abstract

**Introduction:**

In horses, heat exposure modulates the hypothalamic-pituitary-adrenal axis, autonomic nervous system, and hypothalamic-pituitary-thyroid axis to maintain body temperature and prevent excessive heat accumulation. However, during strenuous exercise under hot and humid conditions, heat production may exceed dissipation, leading to heat stress, anhidrosis, heat stroke, or brain damage.

**Methods:**

Incremental field standardized exercise tests (fSETs) provide a reliable approach to assess training and fitness levels. Six Arabian horses from Italia Endurance Stable and Academy were monitored during fSETs performed under heat stress (HS) and thermoneutral (TN) conditions, with blood samples collected before and after each test. Hematocrit, lactate, and biochemical parameters were measured, and total serum RNA was sequenced. A protein-protein interaction (PPI) network of miRNA targets was constructed and analyzed for Gene Ontology (GO) enrichment.

**Results:**

Lactatemia and hematocrit were significantly higher in HS vs. TN, while alanine aminotransferase, creatinine, and creatine kinase increased in HS POST vs. PRE fSET. Differentially expressed small RNAs included eca-myomir-206, eca-mir-301, eca-mir-3613-3p, eca-mir-142, and eca-mir-144, which were modulated by temperature and exercise. In POST vs. PRE fSET, enriched terms involved transcriptional regulation, glucose and LDL response, intracellular trafficking, cytoskeleton organization, cardiac conduction, ion channels, and immune regulation. In HS POST vs. PRE fSET, enrichment was observed for positive regulation of dendritic cell cytokine production, negative regulation of inflammation, and attenuation of oxidative stress-induced apoptotic signaling.

**Discussion:**

This study aimed to investigate the molecular features underlying resilience and adaptation to combined heat- and exercise-induced stress in horses. Overall, our findings indicate that heat amplifies the physiological burden of endurance exercise and alters the molecular mechanisms supporting performance and recovery. Circulating small RNAs may act as early signals for homeostatic restoration and could help elucidate adaptive responses to stress, guiding personalized training strategies.

## 1 Introduction

To maintain homeostasis in a changing environment, both humans and animals have evolved complex physiological systems that allow them to detect and respond to noxious stimuli. These adaptive responses, which involve the neuroendocrine and immune systems, are essential for survival and performance (1, 2). Environmental and non-infectious stressors can activate danger signaling pathways similar to those triggered by infectious agents, leading to innate immune activation and systemic physiological adjustments (3–5). Identifying biomarkers that can objectively monitor these responses is therefore crucial for evaluating animal health and welfare, as well as for understanding the mechanisms underlying adaptive failure and poor performance (6, 7).

Among environmental challenges, heat stress has emerged as one of the most pressing issues due to global climate change. Since the 1950s, the rise in global temperature has accelerated (8) with measurable effects already evident across Europe (9). In southern Italy, for instance, the highest temperature ever recorded in Europe (48.8 °C, Sicily, August 2021) exemplifies the trend toward increasingly frequent heat waves, with the number of days exceeding 35 °C continuously growing (10). Heat stress compromises thermoregulation and homeostasis, affecting animal productivity, fertility, immune competence, and disease susceptibility (11). Understanding how organisms adapt — or fail to adapt — to rising temperatures is therefore critical for ensuring animal welfare and sustainable performance under future climatic scenarios.

Physical exercise represents another potent stressor, sharing many physiological pathways with thermal stress. Intense or prolonged exertion induces systemic responses involving the hypothalamic–pituitary–adrenal (HPA) axis, the autonomic nervous system, and the immune system. Exercise causes transient immunosuppression characterized by leukocytes redistribution (increased neutrophils, decreased lymphocytes) and elevated cortisol, reflecting an acute-phase stress response (12, 13). Cortisol playing a key role in regulating energy availability and modulating the inflammatory cascade, interacting with cytokines such as IL-6 and the interleukin-1 receptor antagonist (IL-1Ra), supporting the maintenance of immune homeostasis during and after exercise (14, 15). Moreover, it has been suggested that the correlation between cortisol and electrophoretic protein pattern modifications induced by exercise may reflect physiological mechanisms aimed at re-establishing homeostasis, as the magnitude of these changes appears insufficient to elicit clinically meaningful alterations (16). Exercise also induces modifications in lipid metabolism, largely driven by hormonal adjustments that facilitate the mobilization and utilization of fatty acids to support muscular energy demand. As shown in athletic horses, circulating lipid fractions may vary according to exercise intensity, metabolic status, and training adaptation, reflecting the dynamic interplay between endocrine responses and substrate availability (17).

Heat exposure activates a series of thermolytic, and compensatory mechanisms aimed at preserving core body temperature. These include the stimulation of the hypothalamic–pituitary–adrenal (HPA) axis and the autonomic nervous system, together with a parallel suppression of the hypothalamic–pituitary–thyroid (HPT) axis. As a consequence, horses exhibit an increase in heart rate and peripheral vasodilation, while metabolic rate is downregulated. Blood flow is preferentially redistributed to the skin to promote heat dissipation, at the expense of perfusion to internal organs (18). Although horses are among the few domestic species that rely heavily on sweating as their primary avenue for evaporative heat loss, their thermoregulatory efficiency is limited by a relatively low surface-to-body mass ratio (1:90–100 m^2^/kg) compared with humans (1:35–40 m^2^/kg), which reduces the effectiveness of heat exchange with the environment. This constraint significantly increases the energetic cost of thermoregulation, especially when combined with physical exercise, which markedly elevates endogenous heat production owing to high muscular activity or prolonged exercise (19). Under demanding environmental conditions, particularly after intense work in hot or hot–humid climates, the rate of heat accumulation may exceed the capacity for dissipation, predisposing horses to heat stress and, in severe cases, to heat stroke accompanied by profound metabolic derangements (20). Maintaining physiological balance during and after exercise performed under heat stress, indeed, requires the coordinated activation of thermoregulatory, endocrine, and antioxidant pathways. Exposure to high temperatures increases metabolic activity and promotes the production of reactive oxygen species (ROS), which, beyond their well-known potential to induce oxidative damage, also act as essential second messengers involved in muscle adaptation and cellular signaling (21).

Investigating how heat and exercise interact offers valuable insight into resilience mechanisms, particularly as future climatic conditions will increasingly expose athletes — human and animal alike — to combined thermal and metabolic stressors (22).

The Arabian horse represents a unique model for studying adaptation to both heat and endurance exercise. Originating from arid environments, this breed has undergone natural and artificial selection for stamina, efficient thermoregulation, and aerobic metabolism. Its relatively homogeneous genetic background makes it a powerful model for exploring complex traits such as resilience to environmental stress (23, 24). Physiologically, horses exhibit exceptional athletic capacity: large intramuscular glycogen stores, high mitochondrial density, and an enhanced oxygen-carrying capacity due to splenic contraction during exertion (25). Their maximal aerobic capacity (VO_2_max) is about 2–2.6 times that of equally sized cattle or highly trained humans (26), reflecting extraordinary cardiovascular and muscular efficiency. Nevertheless, despite these advantages, horses are more prone to heat accumulation than humans because of their lower surface area-to-volume ratio and greater thermal inertia, which slow down heat dissipation (27).

Within equestrian disciplines, endurance racing represents the most demanding challenge in terms of both physical effort and environmental exposure. These long-distance competitions (up to 160 km) require sustained aerobic metabolism, exposing horses to dehydration, electrolyte imbalance, and thermal load that may lead to metabolic collapse, exertional heat illness, or even life-threatening conditions such as rhabdomyolysis and colic (12, 13, 28, 29). For this reason, the discipline is tightly regulated by the National (FISE – Federazione Italiana Sport Equestri) and International Equestrian Federations (FEI) to safeguard equine welfare (30). Despite conditioning programs designed to promote aerobic efficiency (31), prolonged exercise in hot environments can still disrupt homeostasis by overactivating the HPA and thyroid axes and impairing recovery (18, 32)

Given the increasing environmental pressure and the physiological complexity of endurance performance, objective monitoring tools are essential to assess training adaptation, prevent maladaptation, and optimize performance. Incremental field standardized exercise test (fSET) provides a practical, field-based framework to evaluate fitness, physiological response, and recovery dynamics under controlled, realistic conditions (33).

A recent way to investigate molecular changes that reflet organism's response to internal and external stimuli is to detect microRNAs (miRNAs) circulating in the blood. MicroRNAs (miRNAs) are small non-coding RNAs that regulate gene expression at the post-transcriptional level and play essential roles in maintaining cellular homeostasis (34). In horses, as in other mammals, tissue-specific miRNAs contribute to key physiological processes, including muscle remodeling, metabolic regulation, hepatic function, and inflammatory control (35). Beyond their local roles, a proportion of miRNAs is released into the circulation within extracellular vesicles or bound to protein complexes. These circulating miRNAs can be detected non-invasively and provide a systemic “liquid biopsy” particularly valuable in athletic horses, where exercise and environmental heat can simultaneously affect multiple tissues and regulatory pathways (20, 36). Endurance competitions and race efforts have been shown to induce qualitative and quantitative changes in serum or plasma miRNAs, reflecting muscle remodeling, metabolic adaptation, and immune–inflammatory regulation in response to prolonged exercise (5, 37–39). Differences in training status, type of work, and external conditions such as ground surface and weather have also been associated with distinct miRNA signatures, supporting their role as sensitive biomarkers of the physiological response to exercise (40).

This study aimed to investigate the molecular features of resilience and adaptation to the combined stress of heat and exercise in endurance horses, integrating next-generation sequencing (NGS) of circulating small RNAs with hematological and biochemical analyses performed during fSET sessions.

## 2 Materials and methods

Blood samples analyzed in this study were collected as part of a previous research project (33).

### 2.1 Horses enrolled and training

Horses were eligible for inclusion if they underwent, as part of their regular training schedule at Italia Endurance Stables and Academy, Agello (Perugia, Italy), two incremental fSETs per year between October 2018 and February 2022: one during summer (heat stress period [HS], June–August) and one during the rest of the year (thermoneutrality period [TN]). Only horses that had qualified for Concours d'Endurance International (CEI) and raced at a minimum level of CEI^*^ were included (33); six horses fulfilled these criteria. For each horse, all measurements were repeated over two consecutive years, resulting in a total of four fSETs per horse (2 HS and 2 TN).

All horses were stabled at the same training center where fSETs were performed, ensuring uniform environmental and management conditions. Horses were stabled in small paddocks (20 x 20 m approximately) equipped with a 3-side shelters allowing direct outdoor access. Feeding management was standardized and daily quantities were adjusted slightly according to individual body weight. During the out-of-training season, horses received approximately 10 kg/day of first-cut mixed grass hay. During the training and competition seasons, the diet consisted of approximately 10 kg/day of first-cut mixed grass hay and 2–3 kg/day of a mixed-cereal pelleted concentrate, supplemented with a fat source (500 g/day of pelleted rice bran) and powdered vitamin E (50 g/day).

All horses were monitored during fSETs (phase 1: 37 km at 20.8–21.2 km/h; phase 2: 1,400 m at 26.8–27.5 km/h; phase 3: 1,400 m at 30–32.3 km/h) performed by each horse. All fSETs included a warm-up phase consisting of 500 meters at a walk before the fSET. At the end of phase 3, horses underwent 5 min of active recovery at walk, the saddle was then removed, and the horses were sprayed with cold water to simulate the cooling phase of endurance competitions. The heart rate was assessed using a handheld monitor (Polar Equine Healthcheck, Bologna, Italy) and the recovery time was calculated from when the saddle was removed until the heart rate reached 64 bpm (heart rate for entry the vet gate in competition).

### 2.2 Weather data

Weather data for each fSET day were obtained from the weather station sited in Corciano (Perugia, Italy), which is ~10 km north-east from the racetrack. The weather data extrapolated from the website (https://www.ilmeteo.it) were the minimum, maximum, and average environmental temperature and humidity. Environmental conditions were retrospectively assessed using two indices that are based on environmental temperature and relative humidity: the Heat Index (HI = Temperature [°F] + Humidity [RH]) and the Temperature-Humidity Index (THI = 0.8^*^ Temperature [°C] + Humidity [RH]^*^(Temperature [°C]−14.4) + 46.4) (16, 41, 42). The daily mean, minimum and maximum HI and THI values were calculated for the day of sampling and the 7 days before. These metrics provided a comprehensive overview of both acute and cumulative heat stress exposure experienced by the horses during the fSET.

### 2.3 Samples collection

**Figure 1:**
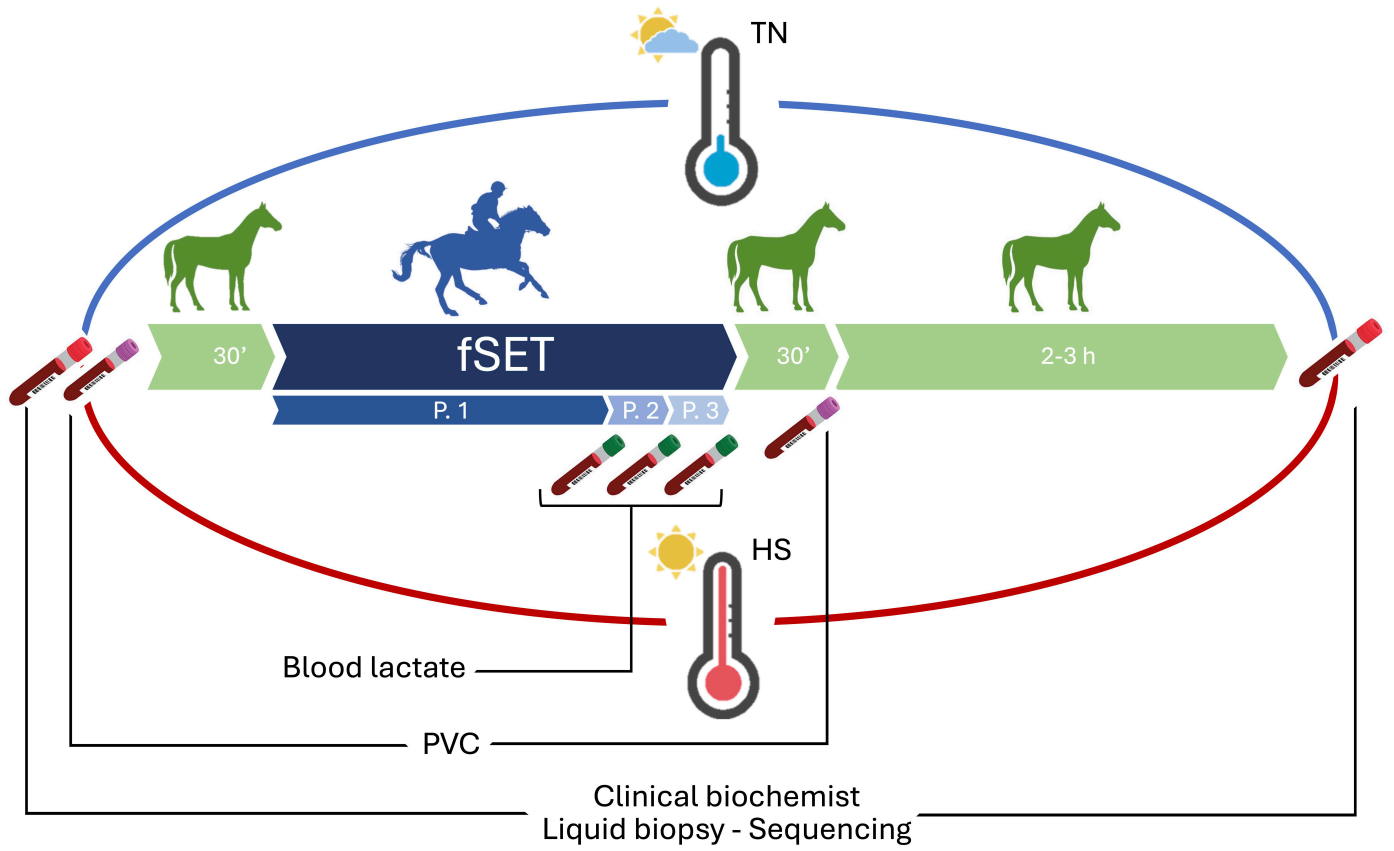
Experimental design and sampling schedule. Six Arabian horses underwent an fSET in both thermoneutrality (blue) and heat stress (red) seasons. Blood samples were collected before (30 min) and after (2–3 h) each fSET for biochemical and liquid biopsy analyses on serum. Packet cell volume (PVC) was evaluated 30 min before and 30 min after fSET. Additional samples were taken at the end of each incremental phase (P.1, P.2 and P.3) to measure blood lactate levels.

Blood samples in the TN and HS, were collected by direct venipuncture from the jugular vein before and after the fSET (Figure 1). Serum was sampled 30 min before and 2–3 h after the fSET, and immediately stored at −80 °C until use for clinical biochemistry and liquid biopsy analysis on circulating small-RNA through Illumina NGS. In particular, the following biochemical parameters were evaluated: urea, glucose, creatinine, lactate dehydrogenase (LDH), glutamic-oxaloacetic transaminase (GOT), alanine aminotransferase (GPT/ALT), direct bilirubin, phosphorus, total bilirubin, albumin, chlorine, creatine kinase (CK), total protein, iron, magnesium. Packet cell volume (PVC) was evaluated on whole blood in EDTA, 30 min before and 30 min after fSET, to assess hematocrit data using a microhematocrit centrifuge (Centrifugal for u-HCT, Dyaset). Moreover, additional samples were taken at the end of each incremental phase (after phase 1, phase 2 and phase 3) to assess blood lactate concentration, which was measured on whole blood in lithium heparin with the stable side dry-slide technology (Chemistry Analyzer IDEXX Catalyst One, Milan, Italy), as shown in Paris et al. (33).

### 2.4 Statistical analysis

All statistical analyses were conducted using R software (version 4.3.0) (43). Data were first checked for completeness, and descriptive statistics were calculated for all variables. For performance and lactate-related parameters (e.g., HCT, Speed_Phase_1–3, Recovery time, Lactate_Phase_1–3, and Lactate_Threshold), comparisons between the two environmental conditions (TN – thermoneutral; HS – heat stress) were performed using paired *t*-tests. For the biochemical dataset, normality was assessed through Shapiro–Wilk tests and Q–Q plots, transforming data as appropriate if not normally distributed. A principal component analysis (PCA) was conducted on the standardized dataset to explore multivariate patterns among experimental groups (TN_PRE, TN_POST, HS_PRE, HS_POST), and the proportion of variance explained by each principal component was reported. Univariate analyses for the same parameters were performed using two-way mixed-effects ANOVA models (*nlme* package), with season (TN and HS) and sampling (PRE and POST fSET) as fixed effects and horse ID as a random effect to account for repeated measures. When significant effects were detected, estimated marginal means were computed using the *emmeans* package, and pairwise comparisons were carried out by applying the Tukey *post-hoc* test. Significant group differences were summarized with compact letter displays (CLD) using the *multcompView* package. Final results are presented as mean ± standard error (SE), with distinct letters indicating statistically significant differences (p < 0.05). Data visualization, including heatmaps, PCA plots, and boxplots, was performed using the *ggplot2* and *ggpubr* packages.

### 2.5 RNA isolation and sequencing

Total RNA was extracted from serum using the miRNeasy Serum/Plasma Advanced Kit (Qiagen, Hilden, Germany), following the manufacturer's protocol. Spike-in sequences were added to the lysis buffer at the beginning of the extraction procedure (2.5 μL of Spike-in solution per 600 μL of serum for each sample) using the QIAseq miRNA Library QC Spike-in Kit (Qiagen, Venlo, The Netherlands), which includes 52 spike-ins phosphorylated at the 5′ end—an essential feature for subsequent library preparation (44). RNA concentration and purity were assessed using a NanoDrop 2000 spectrophotometer (Thermo Fisher Scientific, Waltham, MA, USA), while RNA integrity was evaluated via microfluidic electrophoresis using a Bioanalyzer 2100 (Agilent Technologies, Santa Clara, CA, USA). Library preparation was performed with the QIAseq miRNA Library Kit (Qiagen, Hilden, Germany) according to the manufacturer's instructions, and sequencing was carried out on an Aviti instrument (Element Biosciences, San Diego, CA, USA) using 75 bp single-end reads.

### 2.6 Bioinformatic analysis

Raw sequencing reads, provided in FASTQ format, were first subjected to quality assessment using FastQC (https://www.bioinformatics.babraham.ac.uk/projects/fastqc/). Adapter sequences and low-quality bases were trimmed with Trim Galore v0.5.0 (https://www.bioinformatics.babraham.ac.uk/projects/trim_galore/). The resulting high-quality reads were then aligned using the Bowtie2 aligner (45), following a three-step mapping strategy: initially against the spike-in control sequences used in the experiment; subsequently, unmapped reads were aligned to the miRBase v22.1 hairpin database (http://www.mirbase.org); and finally, any remaining unmapped reads were mapped to the Equus caballus reference genome (EquCab3). After alignment, data normalization was performed with the RUVSeq R package, which estimates unwanted sources of variation based on spike-in read counts, incorporating both season (TN and HS) and sampling time (PRE and POST) as factors in the differential expression model. Differential expression analysis was conducted using DESeq2 (46), applying thresholds of |log_2_ fold change| > 1.0 and a false discovery rate (FDR) < 0.05, corrected for multiple testing using the Benjamini–Hochberg method. A principal component analysis (PCA) plot was generated to visualize sample clustering.

### 2.7 Target gene prediction and functional enrichment analysis

To investigate the potential biological functions of the differentially expressed (DE) miRNAs identified through DESeq2 analysis, their human or murine orthologs were retrieved, when available, from miRBase (http://www.mirbase.org). The most commonly expressed 5p or 3p isoforms were selected and submitted to the miRWalk 3.0 platform (http://mirwalk.umm.uni-heidelberg.de) to obtain a comprehensive list of both predicted and experimentally validated messenger RNA targets. For each miRNA, target sites were categorized according to their location within the transcript:3′UTR,5′UTR, or coding sequence (CDS). Targets were then filtered based on a threshold of at least 70% of DE miRNAs sharing the same target, and the corresponding genes were retained for downstream analyses. To explore potential protein-level interactions, Cytoscape 3.7.1 was used to construct a protein–protein interaction (PPI) network based on data from the IMEx database, which integrates high-confidence, nonredundant interaction information from multiple sources. Network clustering was conducted using the clusterMaker2.0 plugin with the gLay algorithm to identify topological modules, which were subsequently subjected to GO enrichment analysis to determine overrepresented biological pathways. Functional enrichment was performed using the BiNGO plugin in Cytoscape, focusing on Gene Ontology (GO) Biological Process terms. Results were considered statistically significant when meeting a FDR threshold of < 0.05, corrected using the Benjamini–Hochberg method.

## 3 Results

### 3.1 Environmental conditions

According to the weather station data collected during each fSET, environmental temperature and humidity varied consistently between the two periods (Supplementary Table 1).

Under TN fSETs, the average maximum HI was 126.12 ± 14.03, and the average maximum THI was 54.46 ± 9.56, indicating the absence of significant thermal stress. In contrast, during HS fSETs, the average maximum HI reached 150.76 ± 8.52, and the average maximum THI was 75.98 ± 2.78, corresponding to moderate-to-severe heat load conditions.

### 3.2 Blood lactate and hematocrit parameters

Blood lactate concentrations measured at the end of each phase of the fSET were significantly higher in the HS fSETs compared to the TN fSETs. This difference was already evident after Phase 1 (p < 0.001), and remained significant after Phase 2 (*p* < 0.01) and Phase 3 (*p* < 0.05), indicating a greater reliance on anaerobic metabolism under heat stress.

**Figure 2:**
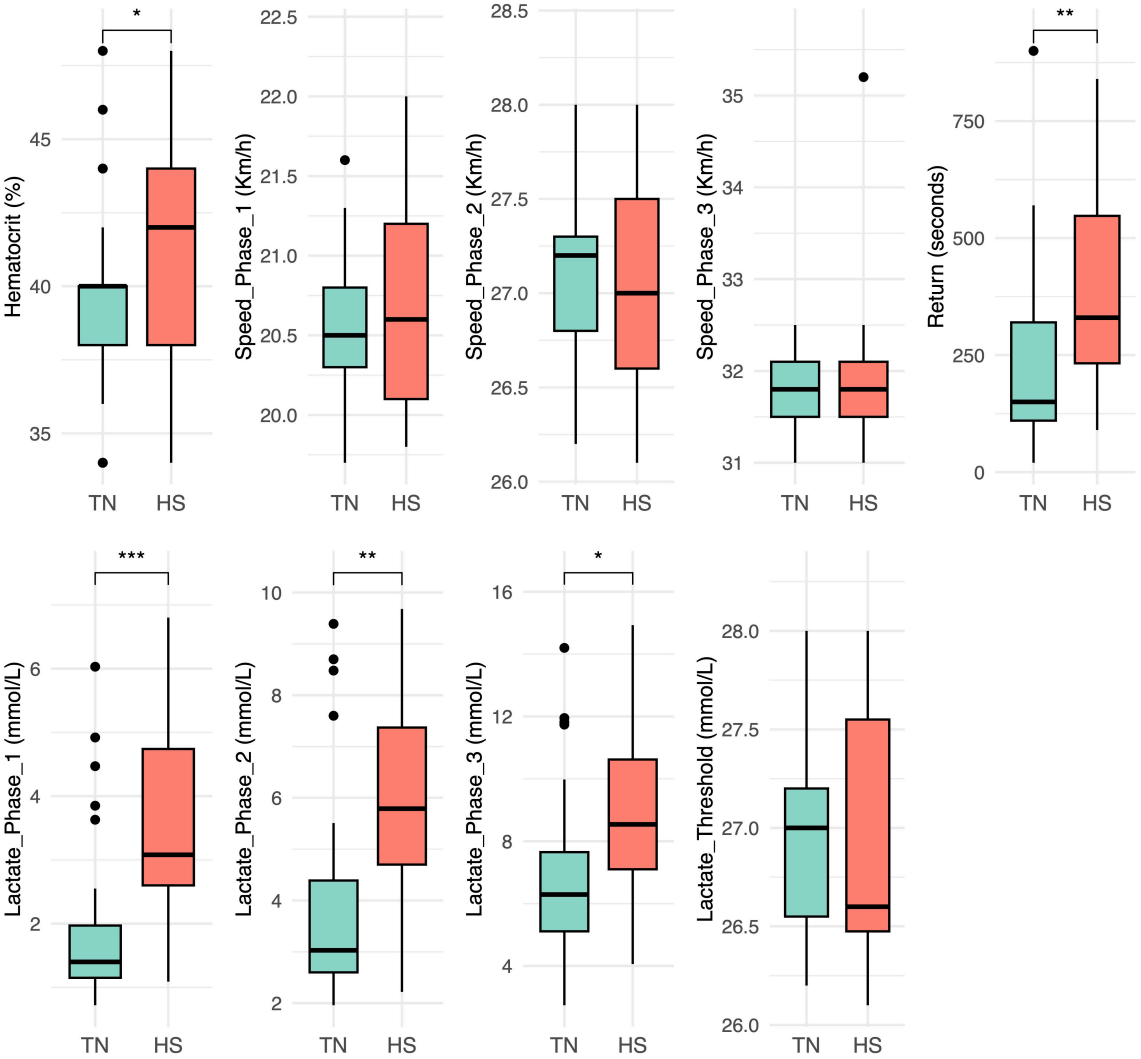
Boxplots showing hematocrit, speed reached in each incremental phase of the fSET (Phase 1, 2, and 3), seconds to return, blood lactate concentrations at the end of each incremental phase of the fSET (Phase 1, 2, and 3) and lactate threshold, comparing thermoneutrality TN (aqua green) and heat stress HS (red) environmental conditions. Statistical significance: ^*^p-value < 0.05, ^**^p-value < 0.01, ^***^p-value < 0.001.

PCV values also differed between conditions. In particular, *post-test* PCV was significantly higher in the HS condition compared to the TN (*p* < 0.05) (Figure 2 and Table 1).

**Table 1:** Numerical values of mean ± standard deviation of thermoneutrality (TN) and heat stress (HS) for hematocrit, speed reached in each incremental phase of the fSET (Phase 1, 2, and 3), seconds to return, blood lactate concentrations at the end of each incremental phase of the fSET (Phase 1, 2, and 3) and blood lactate threshold.

**Parameter**	**TN**	**HS**	**p_value**
Hematocrit (%)	39.21 ± 3.36	41.53 ± 3.91	0.04
Speed_Phase_1 (Km/h)	20.57 ± 0.46	20.72 ± 0.66	0.20
Speed_Phase_2 (Km/h)	27.08 ± 0.48	27.01 ± 0.61	0.70
Speed_Phase_3 (Km/h)	31.77 ± 0.45	31.93 ± 0.97	0.86
Return (seconds)	210.81 ± 172.39	388.75 ± 219.45	0.03
Lactate_Phase_1 (mmol/L)	1.85 ± 1.21	3.62 ± 1.68	< 0.001
Lactate_Phase_2 (mmol/L)	3.87 ± 1.94	5.88 ± 2.09	< 0.001
Lactate_Phase_3 (mmol/L)	6.73 ± 2.62	8.72 ± 2.94	< 0.001
Lactate_Threshold (Km/h)	26.91 ± 0.40	26.93 ± 0.70	0.58

Significant differences between TN and HS are indicated by p-value < 0.05.

### 3.3 Clinical biochemical parameters

**Figure 3:**
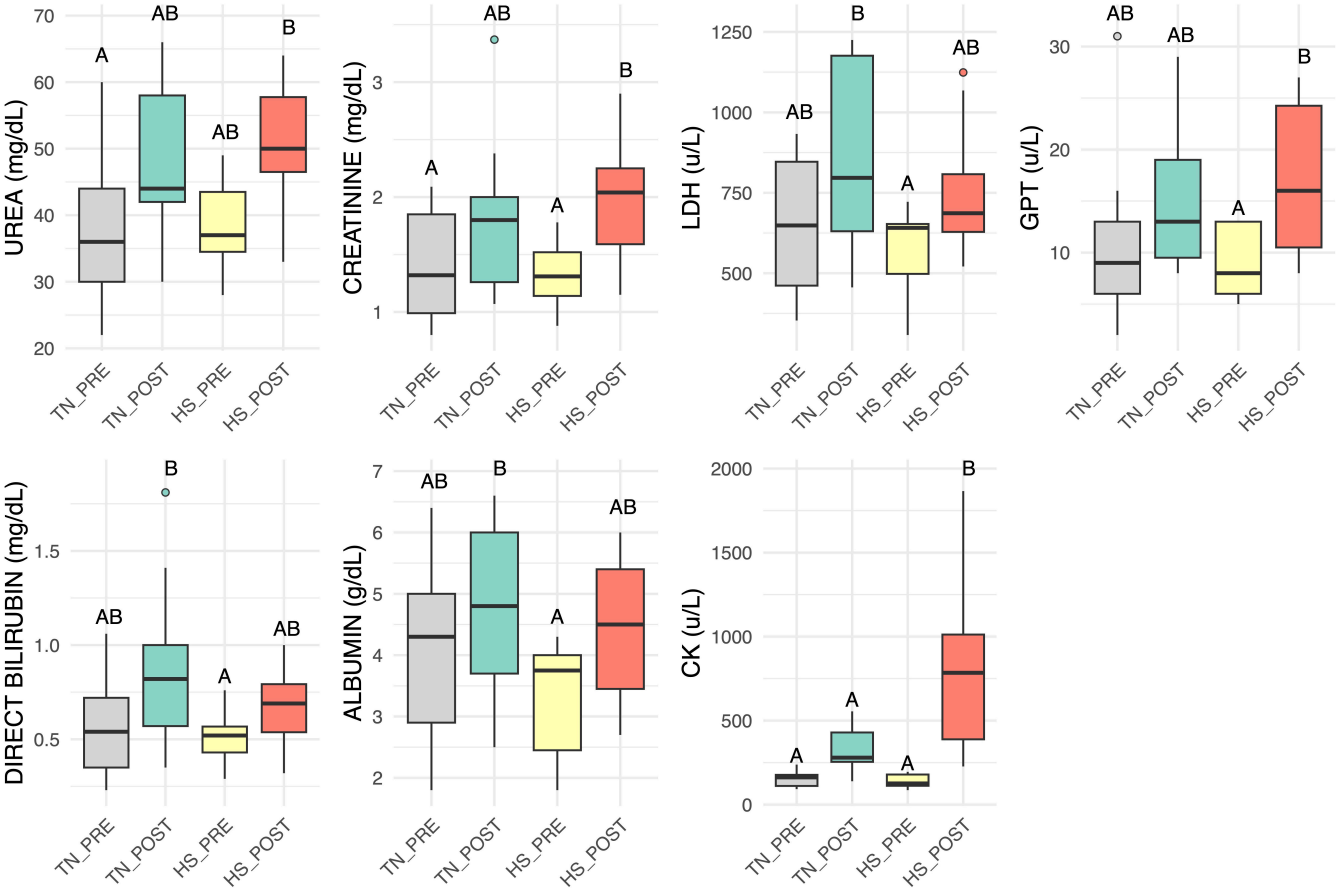
Boxplots of clinical biochemistry parameters displaying the distributions for urea, creatinine, lactate dehydrogenase (LDH), alanine aminotransferase (GPT), direct bilirubin, albumin and creatine kinase (CK) in thermoneutrality TN before (gray) and after (aqua green) fSET and heat stress HS before (yellow) and after (red) fSET. Different letters indicate significant differences (*p* < 0.05).

Blood Endurance exercise elicited clear alterations in several blood biochemical parameters, depending on environmental temperature (Figure 3 and Table 2, box plot of parameters with no statistically significant changes are reported in Supplementary Figure 1). Serum creatinine concentrations significantly increased during the HS period following fSET compared to pre-fSET values, suggesting a potential impairment of renal function. However, the simultaneous elevation in GOT levels may indicate that muscle tissue was also affected by stress, since both creatinine and GOT are largely derived from skeletal muscle (47). Notably, CK levels, a much more muscle-specific marker, rose sharply after exercise in the HS fSET, confirming increased muscular strain or damage, while albumin slightly increased post-exercise, likely reflecting hemoconcentration. Magnesium (Mg) showed a trend toward higher post-test values under HS, though this did not reach statistical significance. Glucose levels were elevated in the HS post-fSET, though this did not reach significance, potentially driven by heightened gluconeogenesis or stress-induced hyperglycemia. The same trend was observed for the LDH, suggesting increased muscle turnover post-exercise in heat.

**Table 2:** Numerical value of means ± standard errors for urea, creatinine, lactate dehydrogenase (LDH), alanine aminotransferase (GPT), direct bilirubin, albumin and creatine kinase (CK) in thermoneutrality TN before (gray) and after (aqua green) fSET and heat stress HS before (yellow) and after (red) fSET.

**Parameter**	**TN_PRE**	**TN_POST**	**HS_PRE**	**HS_POST**
UREA (mg/dL)	38.23 ± 2.76 (A)	48.31 ± 2.76 (AB)	38.40 ± 3.14 (AB)	50.20 ± 3.14 (B)
GLUCOSE (mg/dL)	99.73 ± 9.90 (A)	113.04 ± 9.90 (A)	81.85 ± 10.86 (A)	106.15 ± 10.86 (A)
CREATININE (mg/dL)	1.38 ± 0.14 (A)	1.78 ± 0.14 (AB)	1.32 ± 0.17 (A)	2.00 ± 0.16 (B)
LDH (u/L)	646.92 ± 67.43 (AB)	861.58 ± 67.43 (B)	558.33 ± 77.86 (A)	752.13 ± 82.59 (AB)
GGT (u/L)	14.99 ± 2.32 (A)	18.30 ± 2.32 (A)	13.13 ± 2.56 (A)	16.13 ± 2.56 (A)
GOT (u/L)	286.62 ± 28.25 (A)	351.67 ± 29.40 (A)	241.44 ± 33.95 (A)	267.30 ± 32.21 (A)
GPT (u/L)	11.31 ± 2.18 (AB)	16.59 ± 2.33 (AB)	9.37 ± 2.47 (A)	17.60 ± 2.38 (B)
DIRECT BILIRUBIN (mg/dL)	0.59 ± 0.08 (AB)	0.84 ± 0.08 (B)	0.51 ± 0.09 (A)	0.67 ± 0.09 (AB)
PHOSPHORUS (mg/dL)	4.13 ± 0.53 (A)	3.70 ± 0.53 (A)	3.41 ± 0.60 (A)	3.38 ± 0.60 (A)
TOTAL BILIRUBIN (mg/dL)	1.83 ± 0.29 (A)	2.46 ± 0.29 (A)	1.56 ± 0.33 (A)	2.43 ± 0.32 (A)
ALBUMIN (g/dL)	4.04 ± 0.38 (AB)	4.81 ± 0.38 (B)	3.37 ± 0.43 (A)	4.48 ± 0.43 (AB)
CHLORINE (mEq/L)	102.33 ± 6.38 (A)	108.92 ± 6.38 (A)	90.00 ± 7.37 (A)	104.70 ± 6.99 (A)
CK (u/L)	160.61 ± 72.60 (A)	347.76 ± 75.31 (A)	149.40 ± 85.40 (A)	815.39 ± 81.46 (B)
TOTAL PROTEIN (g/dL)	7.77 ± 0.62 (A)	8.74 ± 0.64 (A)	7.19 ± 0.70 (A)	8.72 ± 0.70 (A)
IRON (ug/dL)	207.70 ± 21.11 (A)	238.28 ± 21.11 (A)	165.11 ± 25.49 (A)	204.07 ± 25.61 (A)
MAGNESIUM (mg/dL)	2.02 ± 0.22 (A)	2.55 ± 0.25 (A)	1.77 ± 0.25 (A)	2.62 ± 0.29 (A)

Statistical significance levels from post-hoc Tukey pairwise comparisons are indicated with letters.

### 3.4 Sequencing results

The sequencing of small RNAs produced an average of over 12 million reads per sample, which were first filtered out by eliminating those of poor quality and then aligned to the microRNA database (miRbase-22) to obtain the most in-depth information for this type of small RNA (Supplementary Table S2). On average, 10 % of cleaned reads were uniquely aligned to miRBase, and an additional 5% were retained after genome mapping. Exploratory analysis of count data is shown in Supplementary Figures S2A, B, where it is possible to observe a not very clear division between the four experimental groups, confirming the result of PCA analyses for hematological data.

#### 3.4.1 miRNA differential expression profiling

Blood Following normalization with DESeq2, a curated dataset of expressed circulating miRNAs was used for differential expression analysis across multiple comparisons designed to capture both exercise- and heat-related effects.

Specifically, three pairwise comparisons were performed:

1. Overall Pre- vs. Post-fSET comparison, including all samples regardless of environmental condition, to identify miRNAs modulated by physical effort *per se*;
2. Pre- vs. Post-fSET in TN, to explore molecular adaptations to exercise under optimal thermal conditions;
3. Pre- vs. Post-fSET in HS, to assess how high environmental temperature influences exercise-induced molecular responses.

A total of 29 miRNAs were detected as significantly differentially expressed (FDR < 0.05, |log_2_FC| ≥ 1.0) across the different contrasts. Among these, 23 miRNAs were modulated by exercise (Overall POST vs. PRE), while 6 and 5 were specific to TN and HS fSET conditions, respectively.

All significant miRNAs were either upregulated or downregulated in the POST-exercise groups compared with their respective controls, suggesting distinct transcriptional programs underlying the physiological responses to exercise, heat stress, and their combined effects at the circulating miRNA level (Table 3).

**Table 3:** Differentially expressed miRNAs among comparisons: post and pre fSET (POST vs. PRE), post and pre fSET in thermoneutrality (TN_POST vs. TN_PRE) and in heat stress (HS_POST vs. HS_PRE) conditions.

**miRNA**	**POST vs. PRE log2FC**	**POST vs. PRE FDR**	**TN_POST vs. TN_PRE log2FC**	**TN_POST vs. TN_PRE FDR**	**HS_POST vs. HS_PRE log2FC**	**HS_POST vs. HS_PRE FDR**
eca-let-7c	−1.10	2.98E-02				
eca-let-7f-2	−1.48	6.40E-03				
eca-let-7g	−1.13	2.56E-02				
eca-mir-101-2	−1.23	1.32E-02				
eca-mir-106°	−1.45	1.81E-02				
eca-mir-129°		1.94	2.38E-02		
eca-mir-1388	−1.27	2.80E-02				
eca-mir-142	−2.52	2.06E-04			−3.26	8.57E-03
eca-mir-144	−2.30	1.31E-03			−3.07	2.34E-02
eca-mir-148b	−1.07	4.86E-02				
eca-mir-16-1	−1.05	3.16E-02				
eca-mir-16-2	−1.17	2.56E-02				
eca-mir-1912	−1.15	9.24E-04	−1.17	4.59E-02		
eca-mir-205	1.27	2.84E-02				
eca-mir-206-2	4.96	4.58E-12	2.66	2.23E-02	5.69	4.94E-09
eca-mir-214	1.03	1.33E-02				
eca-mir-301a		−1.46	3.61E−02		
eca-mir-30c	−1.63	8.13E-03				
eca-mir-33°	−1.61	1.29E-02				
eca-mir-3613	−1.25	7.08E-04	−1.21	4.90E-02		
eca-mir-374a	−1.65	8.13E-03				
eca-mir-545	−2.07	9.70E-03				
eca-mir-590	−1.55	6.70E-03				
eca-mir-885						
eca-mir-8942					1.28	2.33E-02
eca-mir-9102					
eca-mir-9135					1.72	1.44E-02
eca-mir-93	−1.75	3.73E-03				
ENSECAG00000026103	−1.01	2.71E-02				

Expression differences are reported as log2 Fold Change (Log2FC) and statistical significance as false discovery rate (FDR).

#### 3.4.2 Target and functional enrichment analysis

**Figure 4:**
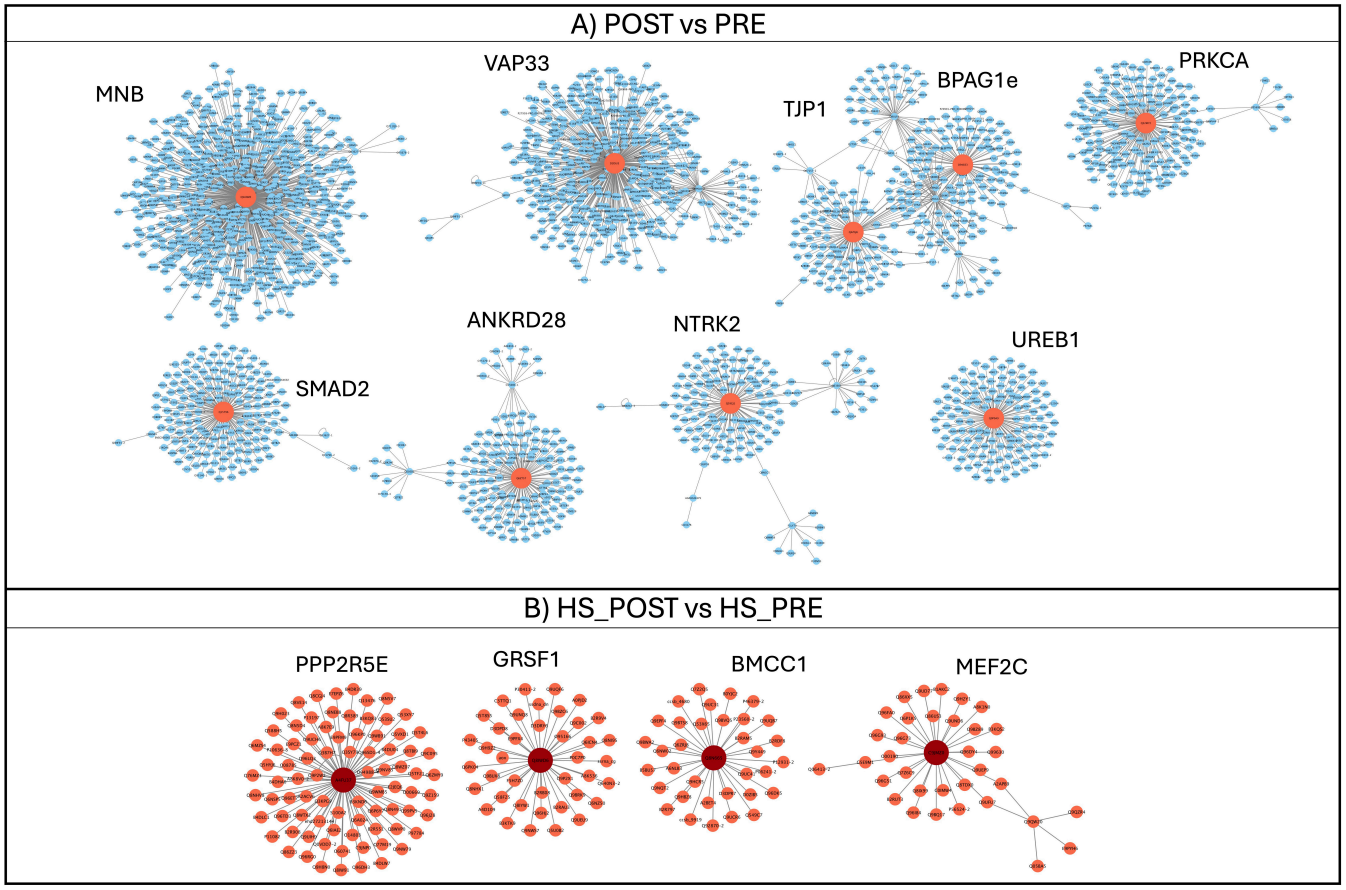
Clusters generated from PPI interaction networks of target genes retrieved for downregulated miRNAs of POST vs. PRE **(A)** and HS_POST vs. HS_PRE **(B)** comparisons. Central nodes of each cluster identifying hub proteins are indicated. Node labels indicate Uniprot identifier assigned by the visualization software while for clusters the gene names of to the central hub of each module is highlighted; for example, the cluster labeled “MNB” corresponds to the node internally identified as UniProt entry Q9UNM5.

Blood Target genes of DE miRNAs were retrieved for all comparisons; however, downstream analysis was only possible for downregulated miRNAs in the POST vs. PRE (Supplementary Table S3) and HS_POST vs. HS_PRE (Supplementary Table S4) comparisons, as no common target genes were identified for the upregulated miRNAs or for the TN comparison. The functional enrichment analysis of miRNA targets allowed us to focus on the heat load associated with the exercise. After target retrieval, the PPI networks generated were composed of 2,718 and 276 nodes in the overall POST vs. PRE and HS_POST vs. HS_PRE downregulated miRNAs, respectively (Figures 4A, B). In both networks, clusters were organized around hub proteins, which represented the most connected nodes within each module and delineated the main functional cores emerging from the analysis. In the POST vs. PRE network, several large and compact clusters were detected, reflecting broad interaction patterns around central hubs. Conversely, the HS_POST vs. HS_PRE network showed fewer modules, suggesting a more focused reorganization of interactions in response to heat stress. In detail in the POST vs. PRE comparison (Figure 4A), the largest and most connected hubs included protein (encoded by target genes) such as MNB, VAP33, BPAG1e, TJP1, and PRKCA, all involved in cytoskeletal dynamics, vesicle transport, and signal transduction, suggesting a broad cellular reorganization following exercise. Additional hubs such as SMAD2, ANKRD28, NTRK2, and UREB1 pointed to regulatory roles in transcriptional signaling and cellular stress responses. In contrast, the HS_POST vs. HS_PRE network (Figure 4B) was dominated by fewer but more specialized hubs (PPP2R5E, GRSF1, BMCC1, MEF2C), reflecting processes related to transcriptional control, RNA processing, metabolic regulation, and pathways typically engaged during heat-stress recovery.

Indeed functional analysis of target clusters derived from downregulated miRNAs after the fSET, highlighted enriched processes related to transcriptional regulation, energy metabolism, insulin signaling, cytoskeletal remodeling, and cellular trafficking, as well as key kinase signaling pathways (e.g., PI3K/Akt, MAPK). These processes are known to support post-exercise recovery and tissue remodeling, including responses to oxidative and mechanical stress. Additionally, immune modulation and type I interferon pathways were enriched, reflecting the balance between activation and resolution of inflammation in response to prolonged exertion. The complete list of GO-enriched terms generated from the genes contained in each cluster can be read in Supplementary Table S5.

Focusing on the HS comparison, the differential expression of specific miRNAs, particularly the downregulation of eca-mir-142 and eca-mir-144, pointed to altered regulation of the JAK/STAT signaling pathway, which may result in increased inflammatory responses under thermal stress. Functional terms derived from clusters analyses included the regulation of transcription in response to stress, apoptotic signaling, cytokine production, and the negative regulation of oxidative stress-induced pathways. All GO enriched terms generated from the genes contained in each cluster are reported in Supplementary Table S6. These results suggest that heat exposure can compromise the physiological adaptation to exercise, possibly increasing susceptibility to systemic inflammation and impairing recovery, particularly through impaired control of stress-responsive and immunoregulatory mechanisms.

## 4 Discussion

This study aimed to explore the physiological and molecular mechanisms underlying resilience and adaptation to combined heat and exercise stress in Arabian endurance horses. First, HI and THI were calculated in order to identify clearly distinct environmental contexts chosen to perform fSET. Based on this environmental characterization, subsequent analyses compared hematological, biochemical, and molecular parameters between TN and HS conditions were performed to elucidate how heat load modulates exercise-induced responses in endurance horses. By integrating these analyses, we characterized both systemic and molecular responses during incremental fSETs performed in thermoneutral and heat stress conditions.

The results demonstrate that environmental heat amplifies exercise-induced stress at multiple biological levels — metabolic, muscular, and immunological — while simultaneously altering the molecular regulatory landscape captured by circulating miRNAs. Together, these findings provide an integrative view of how the Arabian horse, a naturally heat-adapted breed, manages the physiological burden of endurance exercise under rising environmental temperatures.

### 4.1 Physiological and biochemical responses to exercise and heat stress

The combined evaluation of hematological and biochemical variables provides a coherent picture of how endurance horses adjust to the dual challenge of exercise and elevated environmental temperature. During the HS fSET, higher packed cells volume values together with increased creatinine concentrations point toward a more pronounced fluid shift and hemoconcentration than in thermoneutrality (48), likely reflecting greater sweat losses and redistribution of blood flow toward the skin to support heat dissipation. These modifications indicate that, even at comparable workloads, heat exposure imposes a stronger cardiovascular and hydration strain (22). Otherwise, the increase in packed cells volume after exercise can be attributed to physiological adaptations aimed at supporting the elevated oxygen demand of the muscle (16).

Metabolic responses further differentiate between the two environmental conditions. It has been reported that exercise in high heat and humidity triggers hormonal adaptations in horses that help restore homeostasis and reduce metabolic and immune stress (14, 16). In the present study, blood lactate concentrations were significantly higher at the end of all phases of the fSET during the HS period, indicating a greater reliance on anaerobic pathways when exercise is performed in the heat. This pattern suggests that aerobic efficiency is reduced under HS, possibly because part of the cardiac output is diverted to the skin for thermoregulation, limiting oxygen delivery to working muscles. In line with this interpretation, the post-exercise increase in creatine kinase (CK) observed under HS supports the dual pattern of metabolic and structural stress: anaerobic metabolism coupled with micro-muscle damage (49). Such a profile was observed in endurance horses and human athletes in tropical environments, where persistent CK and AST (GOT) elevations mark incomplete recovery and cumulative tissue strain (50). Although lactate dehydrogenase (LDH) did not reach significance, its trend toward elevation supports an increased turnover of muscle tissue. These findings are consistent with previous reports describing exacerbated oxidative stress and mitochondrial dysfunction when exercise is performed in heat conditions, contributing to exertional heat illness (EHI) and post-exercise fatigue (51, 52).

The pattern of liver- and kidney-associated biomarkers further contributes to characterizing how horses respond to exercise under heat stress. In our study, AST (GOT) increased only as a post-exercise trend and did not reach significance. This behavior is consistent with its known kinetic profile, as AST typically peaks 24–48 h after intense exercise and remains elevated longer than CK in horses. The slight post-exercise rise observed here may therefore reflect the very early phase of muscle-related enzyme release rather than a sign of hepatic injury (49). In contrast, ALT (GPT) and GGT showed significant increases exclusively under heat stress, suggesting a mild hepatic adaptation to thermal and metabolic load rather than structural liver damage. These enzymatic shifts are compatible with transient hepatocellular membrane stress (ALT) and with increased metabolic or cholestatic activity (GGT) (49, 53, 54), both of which have been described as part of the physiological adaptation to combined thermal and metabolic load rather than indicators of structural hepatic damage (55). This interpretation aligns with previous reports describing mild, reversible alterations in liver enzymes after prolonged work or environmental heat exposure (21).

Together, these data highlight that identical exercise intensity performed by the same horse elicits a stronger circulatory and metabolic strain when performed in hot conditions compared to thermoneutrality conditions, as demonstrated previously in racehorses (26) and emphasizing the critical importance of temperature-adjusted training and hydration strategies to safeguard equine welfare (51).

Renal-related markers followed a similar pattern of functional adaptation. Elevated urea and creatinine values are consistent with increased protein catabolism, dehydration, and possible reduced renal perfusion during exercise (48, 56). The rise in creatinine observed under heat stress may not solely reflect renal strain but could also be influenced by acute muscle creatine turnover and prerenal hemoconcentration, as previously documented in exercising horses with reduced plasma volume (49). In hot conditions, the likelihood of exercise-induced intravascular hemolysis is also higher due to oxidative stress and mechanical trauma to erythrocytes; this mechanism is compatible with literature reporting increased indirect bilirubin and circulating free hemoglobin in endurance horses following strenuous exertion (54).

Taken together, the coordinated pattern emerging from the hematological, muscular, hepatic, and renal indicators shows that heat amplifies the systemic load imposed by exercise, engaging multiple physiological pathways in a dynamic yet reversible adaptive response aimed at maintaining homeostasis under environmental stress. This integrated framework provides the biological context needed for interpreting the molecular signatures revealed by circulating miRNAs.

### 4.2 Molecular adaptation to exercise under thermoneutral conditions

Under thermoneutral conditions, exercise triggered a coordinated yet controlled modulation of circulating miRNAs associated with muscle adaptation, metabolic remodeling, and immune regulation. The overall *post vs. pre- fSET* and TN *post vs. pre-exercise* comparisons revealed that most differentially expressed miRNAs were downregulated, reflecting a physiological rather than pathological response aimed at restoring homeostasis after exertion.

Among these, eca-miR-206 emerged as one of the most robustly modulated myomiRs in multiple comparisons. miR-206 plays a crucial role in skeletal muscle regeneration, satellite cell activation, and fiber remodeling following exercise (57, 58). Its upregulation in TN comparison suggests activation of anabolic and repair pathways supporting muscle plasticity. Interestingly, the persistent increase of miR-206 under heat stress (HS *post-exercise* vs. HS *pre-exercise*) may instead indicate an adaptive or compensatory response to heat-induced muscle damage and oxidative stress, as previously suggested in both equine and human studies (37, 38, 57).

Conversely, eca-miR-301a was downregulated after exercise in TN. This miRNA is released during endoplasmic reticulum (ER) stress and regulates oxidative damage and apoptosis through KLF7 and LRIG1 signaling (59, 60). Its suppression after exercise may therefore represent a protective adjustment, limiting pro-apoptotic signals and supporting recovery. Similarly, miR-129a, which influences intestinal epithelial integrity and Toll-like receptor signaling, increased after exercise, suggesting reinforcement of barrier and immune balance under metabolic stress (61). The downregulation of miR-1912, involved in the modulation of oxidative stress and apoptosis (62), may reflect a limited degree of cellular stress under TN conditions.

Functional enrichment analysis of these miRNAs' target genes revealed pathways linked to *transcriptional regulation, energy and lipid metabolism, insulin signaling* (PI3K/Akt, GLUT4 translocation), and *cytoskeletal remodeling*—all processes crucial for efficient muscle contraction, metabolic flexibility, and post-fSET recovery (63–65). The observed modulation of insulin-responsive signaling (e.g., PI3K/Akt, GLUT4 translocation) further supports the concept of enhanced glucose uptake and utilization in skeletal muscle, consistent with studies in both humans and equine athletes post-exercise (66, 67). The putative activation of immune-modulatory and anti-inflammatory networks (e.g., TLR signaling, IL-10 expression) suggests a finely tuned response that balances repair and immune tolerance (68). Altogether, these molecular responses indicate that endurance exercise under thermoneutrality elicits a well-coordinated adaptation characterized by muscular remodeling, efficient energy use, and minimal oxidative or inflammatory damage (69–72).

### 4.3 Combined effects of heat and exercise: altered regulation of stress and immune pathways

In contrast, exercise in HS conditions induced a distinct molecular signature characterized by both compensatory and dysregulated responses. Several miRNAs that were adaptively modulated in TN became either suppressed or abnormally upregulated in HS, reflecting the interplay between thermoregulatory strain and cellular stress.

miR-206 remained consistently upregulated after HS exercise, reinforcing its central role in skeletal muscle repair and oxidative stress buffering. However, eca-miR-142 and eca-miR-144 were significantly down-regulated during the hot period, pointing to altered regulation of antioxidant and immune pathways. miR-144 is a known negative regulator of NRF2, the master transcription factor governing antioxidant defense (73–75). Its suppression in HS horses may therefore enhance NRF2 levels, promoting cytoprotection and redox homeostasis. This adaptive response could also help preserve epithelial integrity, which is often compromised under heat and exertional stress (76–79).

Similarly, miR-142, predominantly expressed in hematopoietic cells, modulates immune activation and epithelial barrier homeostasis (80–82). Its downregulation after HS exercise may serve to limit inflammation and support epithelial recovery, as shown in models where miR-142 inhibition enhances barrier function and reduces colitis severity (83). The coordinated suppression of miR-144 and miR-142 thus suggests a protective molecular strategy enhancing antioxidant and barrier defenses while controlling systemic inflammation in endurance horses exposed to heat.

Another interesting candidate, eca-miR-3613a, although it was significantly modulated in overall and thermoneutral comparison, displayed elevated basal expression during HS, even before exercise, implying a chronic pre-conditioning to thermal load. miR-3613-3p has been linked to the regulation of apoptosis and heat shock protein (HSP) genes such as HSPA1A, via the MAP3K2/p38/caspase-3 pathway (84, 85). Its upregulation may therefore reflect an intrinsic thermotolerance mechanism supporting cellular protection against sustained heat exposure.

Functional analysis of HS-specific DE miRNAs revealed enrichment in transcriptional regulation in response to stress, HIF1A, NF-κB, and FOXO signaling, as well as MAPK, JAK/STAT, and PI3K/Akt pathways (86–89). These networks orchestrate oxidative, inflammatory, and apoptotic processes critical for maintaining homeostasis under combined thermal and physical load. The molecular findings are consistent with the physiological data showing increased CK, bilirubin, and GPT under HS, highlighting the tight integration between molecular stress signaling and systemic biochemical markers of tissue strain.

### 4.4 Integrated interpretation: molecular markers of resilience and thermal strain

Horses exposed to exercise in hot environments must activate a coordinated set of physiological adjustments to maintain homeostasis. Heat stress increases metabolic activity and challenges thermoregulation, requiring a redistribution of blood flow toward the skin, enhanced sweating, and modulation of cardiovascular function to preserve core temperature (18). At the cellular level, the rise in metabolic heat promotes the formation of reactive species that, within physiological limits, contribute to signaling pathways involved in muscular adaptation and the activation of antioxidant defenses (21). During recovery, these thermoregulatory, endocrine, and redox-balancing mechanisms interact to stabilize the internal environment and restore functional equilibrium after thermal load. Understanding how these systemic and cellular processes respond to combined heat and exercise provides the necessary context for interpreting the hematological, biochemical, and molecular patterns observed in the present study. The integrated physiological and molecular analyses, taken together, reveal a dual adaptive pattern. Under thermoneutral conditions, endurance exercise elicits a controlled and beneficial remodeling process characterized by efficient energy metabolism, limited oxidative stress, and coordinated molecular regulation. Under heat stress, however, the same exercise intensity induces metabolic inefficiency, dehydration, and muscular strain, accompanied by transcriptional reprogramming toward antioxidant defense and epithelial protection.

The up-regulation of miR-206 across all conditions identifies it as a robust biomarker of muscular activity and recovery, while the suppression of miR-142 and miR-144 under HS highlights their roles in immune-redox balance and barrier integrity. The behavior of miR-3613a suggests potential chronic adaptation to heat and warrants further investigation as a marker of thermotolerance.

## 5 Conclusions

Overall, these findings underscore that heat amplifies the physiological burden of endurance exercise and modifies the fine molecular tuning that normally sustains performance and recovery. Circulating miRNAs emerge as sensitive, non-invasive indicators of such adaptation processes, offering valuable insights for monitoring training status, preventing heat-related disorders, and improving welfare in equine athletes.

## Data Availability

The data presented in the study are deposited in the NCBI Sequence Read Archive (SRA) repository (https://www.ncbi.nlm.nih.gov/sra), Bioproject accession number PRJNA1354229 (Biosamples from SAMN52941646 to SAMN52941691).
