# Supplementary material for: Omic technology to monitoring resilience and adaptation to exercise and heat stress in endurance horses
Source: Front Vet Sci. 2026 Jan 9;12:1734969. doi: 10.3389/fvets.2025.1734969 (PMC12827092; doi:10.3389/fvets.2025.1734969)
Supplement: Supplementary file 1 [file Data_Sheet_1.docx]

Supplementary Material

**
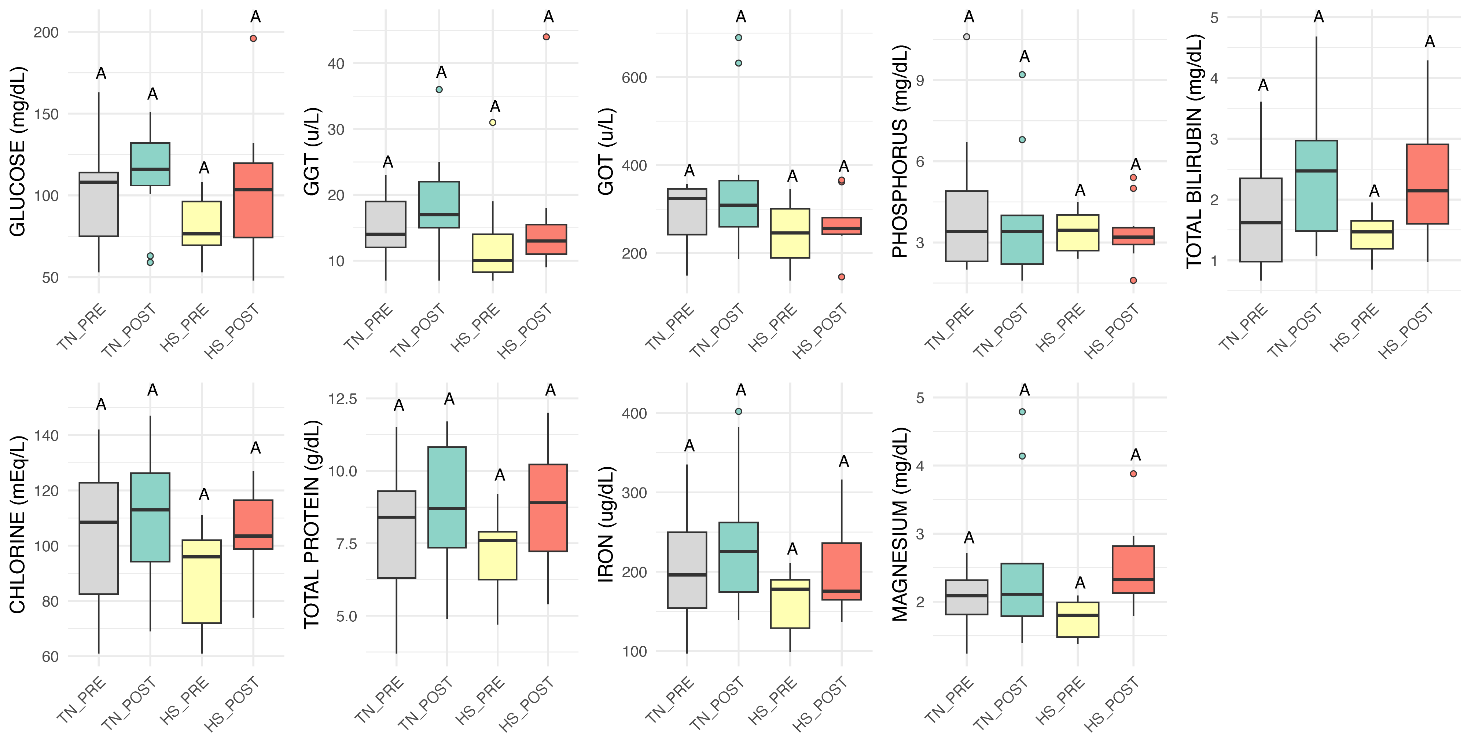
**

**Supplementary Figure 1.** Boxplots of **clinical biochemistry parameters** resulted in no statistically significant changes between thermoneutrality TN **before (**gray**) and after (**aqua green**) fSET** and heat stress HS **before (**yellow**) and after (**red**) fSET.** Statistical significance was assessed with the post-hoc Tukey pairwise comparison test.
