# Supplementary figures and images for: Omic technology to monitoring resilience and adaptation to exercise and heat stress in endurance horses
Source: Front Vet Sci. 2026 Jan 9;12:1734969. doi: 10.3389/fvets.2025.1734969 (PMC12827092; doi:10.3389/fvets.2025.1734969)

**
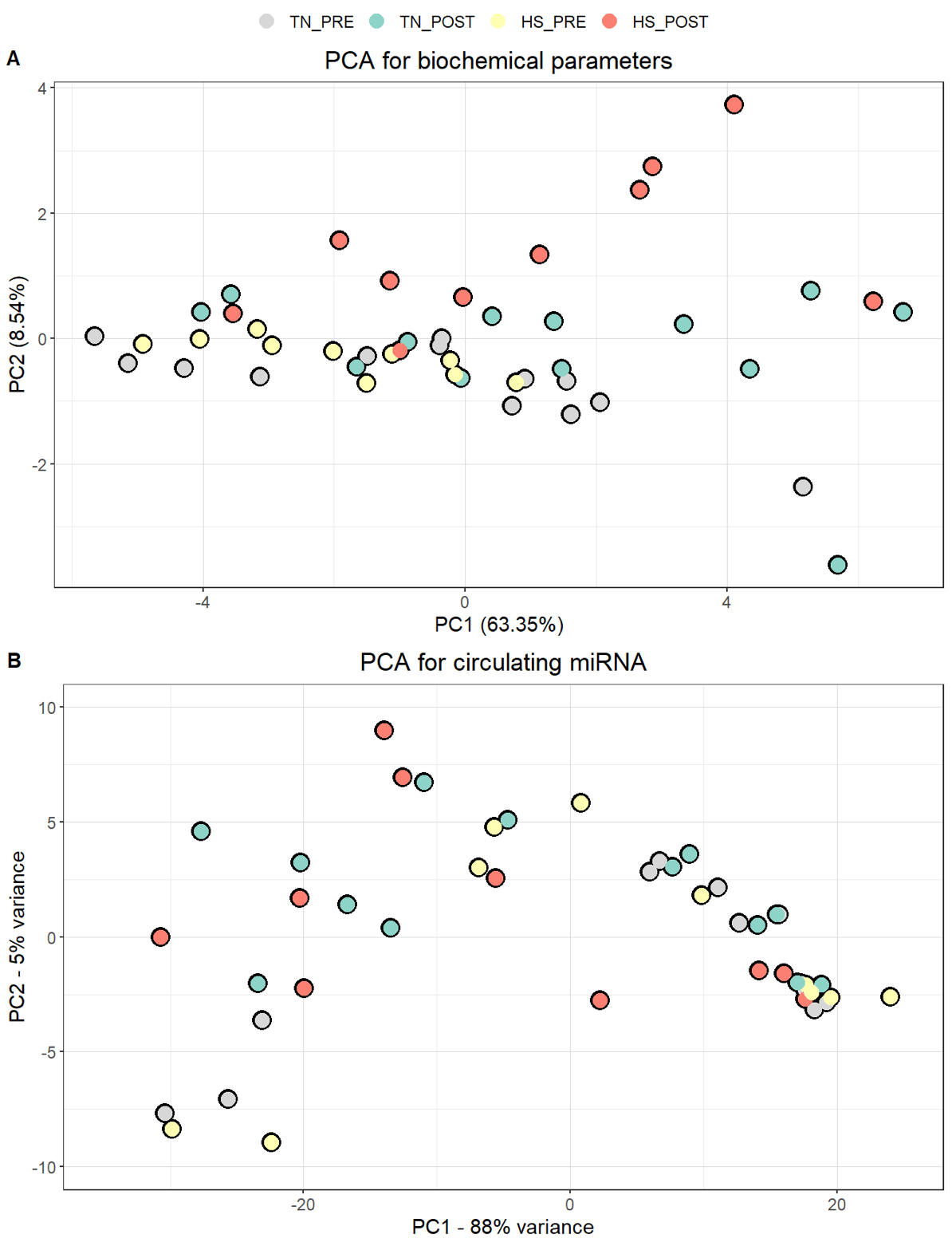
**

**Supplementary Figure 2.** Principal component analysis for biochemical (A) and miRNA data (B).

Supplement: Supplementary file 2 [file Data_Sheet_2.docx]
