# Supplementary material for: Omic technology to monitoring resilience and adaptation to exercise and heat stress in endurance horses
Source: Front Vet Sci. 2026 Jan 9;12:1734969. doi: 10.3389/fvets.2025.1734969 (PMC12827092; doi:10.3389/fvets.2025.1734969)
Supplement: Supplementary file 3 [file Table_1.docx]

Supplementary Material

# Supplementary Tables 1

**Supplementary Table 1.** Mean, minimum and maximum HI and THI for TN and HS sampling.

| **Season** | **Date** | **Mean HI** | **Mean THI** | **Minimum HI** | **Minimum THI** | **Maximum HI** | **Maximum THI** |
| --- | --- | --- | --- | --- | --- | --- | --- |
| **TN** | 11/02/21 | 131.87 | 45.22 | 116.60 | 38.21 | 143.60 | 51.08 |
|  | 12/02/21 | 114.23 | 37.72 | 104.88 | 33.19 | 121.41 | 44.52 |
|  | 13/02/21 | 103.79 | 33.20 | 88.59 | 29.02 | 120.60 | 38.59 |
|  | 14/02/21 | 90.54 | 34.81 | 76.79 | 28.79 | 103.44 | 42.86 |
|  | 15/02/21 | 94.14 | 36.80 | 77.98 | 29.33 | 101.71 | 44.36 |
|  | 16/02/21 | 111.90 | 40.05 | 97.32 | 32.21 | 128.20 | 48.50 |
|  | 17/02/21 | 129.37 | 42.81 | 122.68 | 37.88 | 133.43 | 47.67 |
|  | 03/04/21 | 118.27 | 54.81 | 101.20 | 48.62 | 136.97 | 62.35 |
|  | 04/04/21 | 95.79 | 50.21 | 79.42 | 44.60 | 116.04 | 56.76 |
|  | 05/04/21 | 101.48 | 51.74 | 85.93 | 43.14 | 115.64 | 61.58 |
|  | 06/04/21 | 118.99 | 46.38 | 103.32 | 37.87 | 139.67 | 57.09 |
|  | 07/04/21 | 100.76 | 43.74 | 80.79 | 36.42 | 117.15 | 52.19 |
|  | 08/04/21 | 86.05 | 48.46 | 68.16 | 37.50 | 105.11 | 57.47 |
|  | 09/04/21 | 89.95 | 51.10 | 79.49 | 42.07 | 120.19 | 61.28 |
|  | 16/12/21 | 128.33 | 42.48 | 119.24 | 38.93 | 133.67 | 49.09 |
|  | 17/12/21 | 121.52 | 43.34 | 111.25 | 37.59 | 132.38 | 51.35 |
|  | 18/12/21 | 114.53 | 40.87 | 92.27 | 36.57 | 126.38 | 48.89 |
|  | 19/12/21 | 117.69 | 41.34 | 97.91 | 37.92 | 138.00 | 49.33 |
|  | 20/12/21 | 134.68 | 41.56 | 121.98 | 38.50 | 139.25 | 46.59 |
|  | 21/12/21 | 122.03 | 40.15 | 110.38 | 35.62 | 134.04 | 45.44 |
|  | 22/12/21 | 133.59 | 42.68 | 130.55 | 40.10 | 135.49 | 45.50 |
|  | 05/10/22 | 115.12 | 62.97 | 105.97 | 56.46 | 144.31 | 68.34 |
|  | 06/10/22 | 124.94 | 64.17 | 113.96 | 58.52 | 149.63 | 70.28 |
|  | 07/10/22 | 120.74 | 64.78 | 112.54 | 58.40 | 130.67 | 71.53 |
|  | 08/10/22 | 126.68 | 65.82 | 118.14 | 60.96 | 132.93 | 71.56 |
|  | 09/10/22 | 124.58 | 65.49 | 114.93 | 61.37 | 133.33 | 69.56 |
|  | 10/10/22 | 133.94 | 64.54 | 127.39 | 62.42 | 141.88 | 67.23 |
|  | 11/10/22 | 132.93 | 64.73 | 121.24 | 61.22 | 144.77 | 70.35 |
|  | 08/02/23 | 99.75 | 35.51 | 81.23 | 29.32 | 111.24 | 44.61 |
|  | 09/02/23 | 105.04 | 36.15 | 93.70 | 31.55 | 114.67 | 43.51 |
|  | 10/02/23 | 87.97 | 39.19 | 73.93 | 32.34 | 103.97 | 49.03 |
|  | 11/02/23 | 87.58 | 42.55 | 77.11 | 36.28 | 94.46 | 52.23 |
|  | 12/02/23 | 96.54 | 44.74 | 79.88 | 37.59 | 126.56 | 55.52 |
|  | 13/02/23 | 115.49 | 43.31 | 98.08 | 36.06 | 126.77 | 54.40 |
|  | 14/02/23 | 107.77 | 45.33 | 95.43 | 37.96 | 116.46 | 55.50 |
| **HS** | 30/06/21 | 118.85 | 70.29 | 109.58 | 66.09 | 142.73 | 76.19 |
|  | 01/07/21 | 123.05 | 70.15 | 115.46 | 63.22 | 135.01 | 77.10 |
|  | 02/07/21 | 120.86 | 71.13 | 112.67 | 64.21 | 130.05 | 76.92 |
|  | 03/07/21 | 127.09 | 72.90 | 119.16 | 67.39 | 150.15 | 77.92 |
|  | 04/07/21 | 133.61 | 73.09 | 111.88 | 68.26 | 147.70 | 78.55 |
|  | 05/07/21 | 130.42 | 73.05 | 117.86 | 67.58 | 145.72 | 79.30 |
|  | 06/07/21 | 124.73 | 74.11 | 118.07 | 67.96 | 139.25 | 80.61 |
|  | 16/07/21 | 140.94 | 68.15 | 124.05 | 63.32 | 155.54 | 75.16 |
|  | 17/07/21 | 144.32 | 68.69 | 125.99 | 64.04 | 158.25 | 73.53 |
|  | 18/07/21 | 140.24 | 72.40 | 129.37 | 68.77 | 154.64 | 76.48 |
|  | 19/07/21 | 137.60 | 72.47 | 126.85 | 68.10 | 151.06 | 76.75 |
|  | 20/07/21 | 127.43 | 73.44 | 116.48 | 66.78 | 137.19 | 79.27 |
|  | 21/07/21 | 129.17 | 74.95 | 115.27 | 70.76 | 146.00 | 79.47 |
|  | 22/07/21 | 130.17 | 74.61 | 115.26 | 69.41 | 143.77 | 79.70 |
|  | 31/08/22 | 134.64 | 73.15 | 119.23 | 68.75 | 149.26 | 77.21 |
|  | 01/09/22 | 143.43 | 69.91 | 129.61 | 62.68 | 158.17 | 73.44 |
|  | 02/09/22 | 127.20 | 67.55 | 116.52 | 60.26 | 147.20 | 75.08 |
|  | 03/09/22 | 133.35 | 70.21 | 118.13 | 63.38 | 156.88 | 76.76 |
|  | 04/09/22 | 139.40 | 72.22 | 114.22 | 67.98 | 158.10 | 76.68 |
|  | 05/09/22 | 131.45 | 72.32 | 112.63 | 65.89 | 146.66 | 77.19 |
|  | 06/09/22 | 140.79 | 74.25 | 117.82 | 68.22 | 162.22 | 78.42 |
|  | 01/06/23 | 143.82 | 66.14 | 129.43 | 62.55 | 153.80 | 72.06 |
|  | 02/06/23 | 142.99 | 66.83 | 131.61 | 63.25 | 156.34 | 72.91 |
|  | 03/06/23 | 149.76 | 65.75 | 130.97 | 62.69 | 159.55 | 71.76 |
|  | 04/06/23 | 143.53 | 66.86 | 122.83 | 59.08 | 158.75 | 73.13 |
|  | 05/06/23 | 150.19 | 66.06 | 126.76 | 62.56 | 160.31 | 71.13 |
|  | 06/06/23 | 148.75 | 66.19 | 125.85 | 62.53 | 159.36 | 71.50 |
|  | 07/06/23 | 144.81 | 67.35 | 127.54 | 63.23 | 157.64 | 73.21 |
| **Average ± Standard deviation of TN** |  | 112.53 ± 15.29 | 47.11 ± 10.08 | 99.44 ± 17.70 | 41.27 ± 10.40 | 126.12 ± 14.03 | 54.46 ± 9.56 |
| **Average ± Standard deviation of HS** |  | 135.81 ± 9.02 | 70.51 ± 3.03 | 120.75 ± 6.60 | 65.32 ± 3.02 | 150.76 ± 8.52 | 75.98 ± 2.78 |
