# Supplementary material for: Omic technology to monitoring resilience and adaptation to exercise and heat stress in endurance horses
Source: Front Vet Sci. 2026 Jan 9;12:1734969. doi: 10.3389/fvets.2025.1734969 (PMC12827092; doi:10.3389/fvets.2025.1734969)
Supplement: Supplementary file 4 [file Table_2.docx]

Supplementary Material

# Supplementary Tables 2

**Supplementary table S2.** Sequencing statistics of retained reads after each bioinformatic step.

| Groups | raw reads | reads post umitools | Cleaned reads | % cleaned | unique on miRNAs | % unique on miRNAs | Unmapped miRNAs | unique on genome | % unique on genome |
| --- | --- | --- | --- | --- | --- | --- | --- | --- | --- |
| TN_PRE | 12,320,492.00 | 10,891,008.85 | 7,881,079.15 | 72.36 | 1,051,056.00 | 11.82 | 5,927,812.23 | 302,839.92 | 5.89 |
| TN_POST | 11,375,796.69 | 10,041,804.92 | 6,877,953.54 | 68.49 | 693,819.62 | 9.61 | 5,279,947.38 | 272,748.31 | 5.09 |
| HS_PRE | 13,732,918.1 | 12,238,000.5 | 8,994,237.7 | 73.49 | 1,323,007.1 | 13.717 | 6,360,170.6 | 348,406.3 | 5.66 |
| HS_POST | 12,238,735.9 | 10,929,528.8 | 7,527,266.3 | 68.87 | 590,194.9 | 7.914 | 6,188,913.9 | 281,927.1 | 4.572 |
| Average | 12,416,985.67 | 11,025,085.77 | 7,820,134.17 | 70.81 | 914,519.40 | 10.77 | 5,939,211.03 | 301,480.41 | 5.30 |
